# Machine learning for medication error detection: a scoping review

**DOI:** 10.21203/rs.3.rs-8919709/v1

**Published:** 2026-02-20

**Authors:** Félicien Hêche, Sohrab Ferdowsi, Anthony Yazdani, Sara Sansaloni-Pastor, Douglas Teodoro

**Affiliations:** Department of Radiology and Medical Informatics, University of Geneva, Geneva, Switzerland; Department of Radiology and Medical Informatics, University of Geneva, Geneva, Switzerland; Department of Radiology and Medical Informatics, University of Geneva, Geneva, Switzerland; Actelion Pharmaceuticals Ltd, Basel, Switzerland; Department of Radiology and Medical Informatics, University of Geneva, Geneva, Switzerland

**Keywords:** Machine learning, Medication errors, Scoping review

## Abstract

**Objective:**

Medication errors remain a substantial public health concern, and existing measures, such as workforce training, have achieved only partial success. Advances in data availability and computational methods have led to increasing use of machine learning (ML) to support medication safety. This scoping review synthesizes and categorizes ML-based approaches to medication error detection or prediction.

**Materials and Methods:**

Following Preferred Reporting Items for Systematic Reviews and Meta-Analyses extension for Scoping Reviews (PRISMA-ScR) guidelines, PubMed, Embase, and Web of Science were searched for studies published between 2015 and April 2025. Two reviewers independently performed study selection using predefined eligibility criteria, and data extraction followed a structured extraction framework.

**Results:**

Twenty-two studies met the inclusion criteria. Two dominant ML pipelines were identified. Most studies focused on prescription-related errors, relying on structured clinical data and tree-based models. A smaller group addressed medication-administration errors using unstructured multimodal data, such as images or video, analyzed with neural networks and multi-stage detection pipelines.

**Discussion:**

ML shows substantial potential for medication error detection, particularly in prescription-focused workflows that align well with existing clinical processes. However, the evidence remains fragmented, with limited generalizability, inconsistent labeling, and scarce real-world evaluation. No studies addressed medication errors in clinical research settings, such as clinical trials, despite their distinct workflows and safety implications.

**Conclusion:**

Advancing ML-based medication error detection will require high-quality multicenter datasets, rigorous and transparent validation, and deeper exploration of underused data modalities, including free text.

## 1 Introduction

Medication errors, defined as failures in the treatment process that lead to, or have the potential to lead to, patient harm [1], represent a major public health concern worldwide. These errors are frequent across healthcare systems, affecting a substantial proportion of patients and prescriptions [2, 3, 4], and may occur at any stage of the medication-use process, including prescribing, administration, and dispensing [5, 6, 7]. Their consequences range from minor harm to serious adverse drug events and fatal outcomes [8, 9, 10], while also contributing to increased healthcare costs and prolonged hospital stays [11, 12]. They arise not only in routine clinical care but also in clinical research settings, where complex protocols and investigational products introduce additional risk factors [13, 14].

In response to this critical issue, several health agencies have launched campaigns to promote safer medication practices. The World Health Organization (WHO) introduced the Medication Without Harm initiative [15] and recently issued a policy brief to support its ongoing execution [16]. At the national level, regulatory authorities have similarly prioritized medication safety, establishing dedicated strategies and organizational units to monitor, analyze, and prevent medication errors across clinical care and drug development processes [17, 18, 19, 20].

Various strategies have been implemented to mitigate medication errors, including medication reconciliation [21, 22], staff training [23], automated drug administration systems [24], and packaging or labeling guidelines designed to reduce confusion between look-alike and sound-alike products [25]. However, these interventions often face substantial practical limitations, such as high resource requirements, organizational complexity, training demands, and technical constraints, which can hinder their scalability and sustained implementation [26, 27, 28, 29, 30]. These challenges are particularly pronounced in clinical trials, where investigational products frequently lack finalized packaging or labeling, limiting the applicability of design-focused safety strategies.

In this context, machine learning (ML) has emerged as a promising approach for medication safety, offering the ability to model complex, high-dimensional, and potentially multimodal clinical data that challenge traditional rule-based systems [31, 32]. ML methods can identify subtle patterns associated with prescribing, dispensing, or administration errors and, when integrated into clinical workflows, support real-time decision making and risk prioritization. These capabilities have motivated a growing body of research on ML-based medication-error detection and prediction [33, 34, 35, 36].

A structured synthesis of this literature would be valuable for clinicians seeking to understand which ML tools have been evaluated in medication-safety workflows and for researchers aiming to identify methodological practices and remaining gaps. While prior reviews have examined AI for patient safety more broadly [37], clinical trial risk assessment [38], or medication alert optimization during prescribing [39], no review has yet synthesized ML applications across the full spectrum of medication errors, spanning both clinical care and research contexts.

This scoping review fills this gap by offering the first field-wide synthesis of ML-based medication-error detection, integrating structured and unstructured modalities, supervised and unsupervised methods, and both clinical and simulated environments. The review was conducted according to a pre-specified protocol developed prior to its initiation [40]. This protocol adheres to the Preferred Reporting Items for Systematic Reviews and Meta-Analyses extension for Scoping Reviews (PRISMA-ScR) guidelines [41]. Structured searches were performed across PubMedEmbase and Web of Science, applying predefined inclusion and exclusion criteria to identify relevant studies. Key information, including the type of model, data sources, evaluation methods, and clinical context, was extracted and analyzed through descriptive statistics, visualizations, thematic analysis, and narrative synthesis.

This scoping review is structured as follows. Section 2 describes the methodological protocol, Section 3 presents the main findings, and Section 4 synthesizes these results and discusses their clinical and research implications.

## 2 Methods

The protocol used to conduct our literature review, consists of five key stages: (i) formulation of the research questions; (ii) identification of relevant literature; (iii) study selection; (iv) data charting; and (v) synthesis and analysis of the results. This protocol was preregistered on the Open Science Framework prior to the start of the review (https://doi.org/10.17605/OSF.IO/38SFY).

### 2.1 Stage 1: formulation of the research questions

The aim of this review was to identify how ML methods have been applied to predict or detect medication errors across various medical settings. To achieve this objective, the following research questions were developed:

1. What types of ML methods have been used to predict or detect medication errors?
2. In which medical context have these approaches been applied?
3. What kinds of data sources and modalities have been utilized in these applications?
4. What are the main challenges, limitation, evaluation strategies, and reported outcomes?

### 2.2 Stage 2: identifying relevant literature

As detailed in the review protocol [40], PubMed, Embase, and Web of Science were selected as the primary databases. The search strategy was constructed using the following general query format:

$$\left({\text{Group}}_{1})\;\text{AND}\;({\text{Group}}_{2})\;\text{AND}\;({\text{Group}}_{3})\;\text{AND}\;({\text{Group}}_{4}\right)$$

where each Group_*i*_ corresponds to a predefined set of keywords listed in Table 1. All terms were searched exclusively within the title and abstract fields. The exact keyword search used for each database is provided in Appendix A.1. We considered articles published in English between January 1, 2015, and the date of the search (April 28, 2025).

### 2.3 Stage 3: study selection

Studies were eligible for inclusion if they (i) develop, apply, or evaluate ML methods to predict or detect medication errors; (ii) are related to clinical treatment or research involving human subjects; (iii) are basic research articles published in peer-reviewed journals or conference proceedings, available in full text, and written in English; and (iv) were published between 1 January 2015 and April 28, 2025.

As this review focuses on emerging ML methods capable of modeling complex, non-linear patterns and multimodal data (e.g., clinical text, images), we excluded traditional statistical approaches, such as linear and logistic regression or classical time-series models (e.g., ARIMA [42]), that rely on strong parametric assumptions and are not suited for high-dimensional or unstructured data. In addition, articles that address adverse drug events without explicitly focusing on medication errors were excluded. Works describing methods to prevent medication errors were excluded unless they involved the prediction of such incidents. These inclusion and exclusion criteria are presented in Table 2.

The selection procedure was carried out by two independent reviewers. First, titles and abstracts of the articles were screened to assess their compliance with the eligibility criteria. Studies deemed potentially relevant underwent full-text screening. Those that met the eligibility criteria upon full-text review were included in the final set of studies for the scoping review. For studies where eligibility was unclear or ambiguous, inclusion decisions were resolved through discussion and consensus between the two reviewers.

### 2.4 Stage 4: data charting

For data extraction, we followed the CHecklist for critical Appraisal and data extraction for systematic Reviews of prediction Modelling Studies (CHARMS) [43] framework, including metadata. In addition, data sources, types, features, labels, and dataset size, as well as the specific ML models employed, were extracted. The complete set of extracted information is described in Appendix A.2, Table 5.

### 2.5 Stage 5: synthesis and analysis of the results

The results of the scoping review were synthesized using complementary approaches to provide an overview of research topics and methodological practices across the included studies. The synthesis began with a descriptive summary, reporting numerical indicators such as publication trends, data types, ML models, and clinical contexts. Visualizations, including bar charts and summary tables, were used to present distributions across key categories and to concisely summarize use cases, model performance, and dataset characteristics.

Based on this exploratory synthesis, thematic analysis was conducted to identify recurring themes related to application contexts, methodological challenges, and validation strategies. Finally, a narrative synthesis was used to characterize the scope and nature of ML approaches for medication-error detection, highlighting major research trends, methodological patterns, and gaps in the literature.

## 3 Results

**Figure 1:**
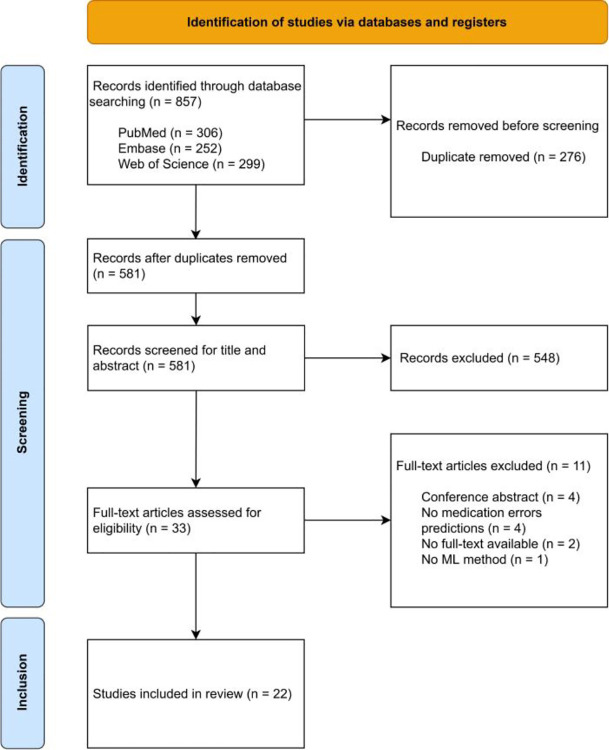
PRISMA flow diagram of the literature search and study selection process.

The initial search retrieved 306 records from PubMed, 252 from Embase, and 299 from Web of Science. Duplicate records were identified and removed from the initial set of 857 articles, using the reference management tool EndNote, resulting in a set of 581 studies. After title, abstract and full-text screening 22 studies met the eligibility criteria (Figure 1). The key characteristics extracted from these studies are summarized in Tables 3 and 4. To maintain clarity and comparability, only standardized elements are reported in the tables, while additional contextual details are synthesized narratively below.

**Figure 2:**
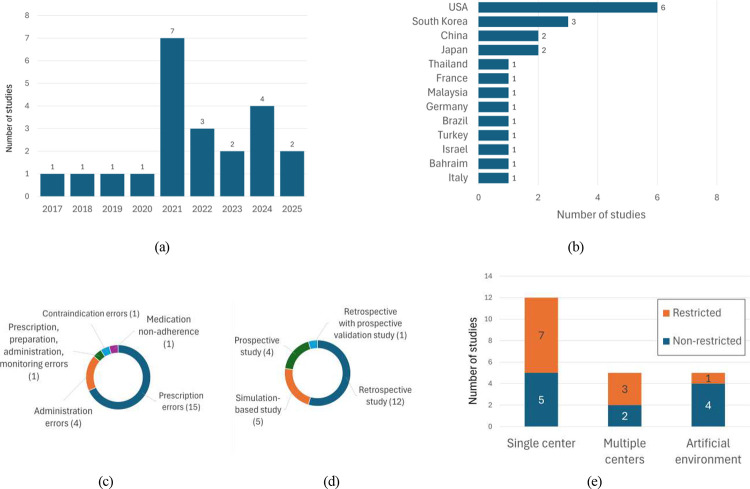
**(a)** annual number of included studies; **(b)** geographic distribution of the publications; **(c)** medication-error types investigated; **(d)** distribution of study designs; **(e)** study context and population restriction.

The temporal distribution of publications (Figure 2a) shows an emergence of research activity from 2017, followed by a marked increase from 2021 onward. Notably, 82% of included studies were published after 2020.

Geographically, the included studies are dominated by Asia and North America (Figure 2b). The United States contributed the largest number of individual research (27.3%), whereas Asia was the most represented continent (54.5%), with contributions spanning South Korea [44, 45, 46], Japan [47, 48], China [49, 50], among other. Europe accounted for only three studies (13.6%) [51, 52, 53], and one investigation originated from South America (4.5%) [54]. No eligible studies were identified from other regions of the world.

### 3.1 Study characteristics and application areas

As shown in Figure 2c, most ML studies for medication errors detection focused on prescription errors (68.2%) [53, 44, 47, 45, 55, 56, 57, 58, 59, 54, 60]. Administration errors were addressed in 18.2% of the studies and typically within narrowly defined or specialized clinical settings [61, 62, 51, 63]. The remaining publications examined specific error types, including medication non-adherence [46], contraindication [50], or multiple error types within a single framework [64].

Retrospective analyses represented over half of the included literature and largely focused on developing ML models for predicting or detecting medication errors (Figure 2d). The remaining studies evaluated existing models or systems [57, 59, 60]. Simulation-based investigations (22.7%) primarily explored prototype systems in controlled or synthetic environments, such as medication-review scenarios [65], or home-based medication-taking simulations [51, 63, 46]. In contrast, prospective studies (18.2%) primarily aimed to develop ML models [61, 62, 64].

More than half of the studies (54.5%) were single-center (Figure 2e). These studies encompassed a wide range of clinical contexts, including general hospital populations [53, 44, 55, 56, 54], specific patient groups [49, 52, 45], or specific prescription categories [47, 48], and specific care units [64, 59]. Five investigations (22.7%) were multicenters, either within specialized environments, [62, 57, 61], or more general outpatient populations [58, 60]. The remaining five studies (22.7%) were conducted in simulated or artificial environments and primarily aimed to develop or test prototype systems, in generic clinical scenarios [65, 50, 51, 46], or specific administration procedures [63]. All identified studies focused on clinical care.

### 3.2 Data used for training and assessing ML

**Figure 3:**
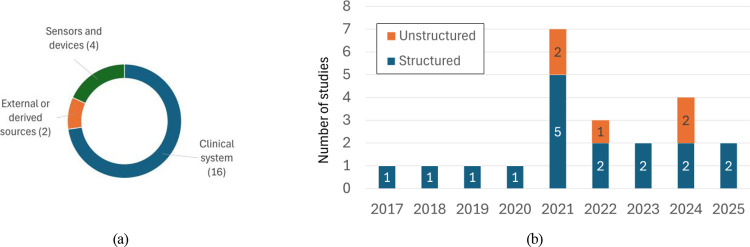
**(a)** data sources used; **(b)** number of studies relying on structured versus unstructured data over time.

Most datasets (72.3%) were extracted from clinical information systems (Figure 3a) and consisted exclusively of structured variables. Several studies supplemented or replaced clinical data with unstructured inputs including videos [62, 46], images [51], and from radio-frequency signals [63]. Among the remaining works, only one processed free-text clinical scenarios using an LLM [65]. Overall, structured clinical data dominated the evidence base, while unstructured data modalities first appeared in studies published from 2021 onward (Figure 3b).

**Figure 4:**
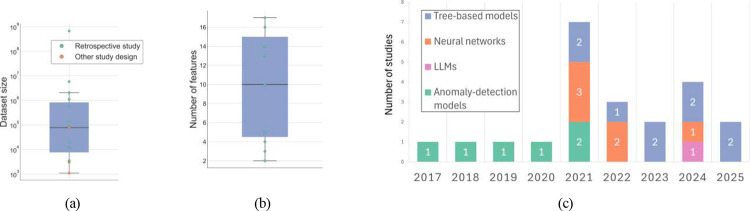
**(a)** distribution of dataset sizes in studies using structured tabular data; when multiple dataset sizes were reported [48, 55, 48], their average was plotted; **(b)** distribution of the number of features across studies using structured tabular data; the study reporting 284 features [50] is excluded from the plot for visualization clarity but included in the boxplot calculation; **(c)** annual number of studies per model category.

Dataset sizes of studies relying on structured data varied substantially (Figure 4a), with retrospective studies, ranging from several thousand records [49, 44] to several million [53, 56]. Prospective and simulation-based studies relied on smaller samples (1,093–82,553 observations) [61, 64]. Works leveraging alternative modalities operated on limited sample sizes [62, 46]. Some studies did not report dataset size, but only the cohort size [58, 60].

Feature dimensionality for structured tabular data was generally low, ranging from 2 [54] to 17 variables [49] (Figure 4b). Common features included patient demographics, clinical characteristics (e.g., diagnoses, laboratory values, vital signs), and prescription attributes such as drug name, dose, frequency, route, and temporal indicators.

In contrast, studies using unstructured data required richer representations: image-based models extracted visual embeddings [51], video-based systems derived object detections and spatiotemporal descriptors [62, 46], and radio-frequency sensors encoded human motion into high-dimensional tensors [63], while some processed free-text inputs directly with an LLM, thereby avoiding explicit feature engineering [65]. Several investigations did not report precise feature details [57, 58, 59, 60].

### 3.3 Model type, prediction outcome, and evaluations

From a modeling perspective, the choice of method most commonly followed the underlying data modality. In studies using structured clinical data, which represented the majority, tree-based ensemble models were the most common [49, 64, 53, 44, 61]. Deep learning applied to structured inputs was less frequent and generally limited to fully connected architectures embedded within hybrid rule–ML pipelines [45]. Studies using unstructured modalities applied neural-network architectures [62, 46, 63, 51], with only one employing LLMs [65]. As illustrated in Figure 4c, earlier work (pre-2021) relied almost exclusively on anomaly-detection paradigms, whereas the adoption of tree-based ensembles and deep neural architectures expanded substantially from 2021 onward.

**Figure 5:**
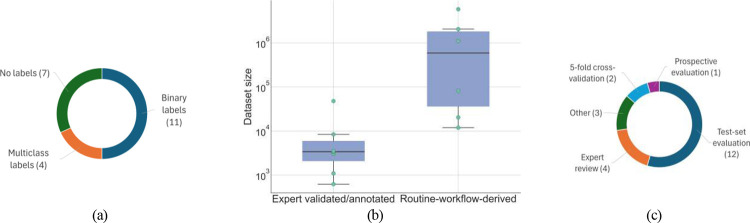
**(a)** type of labels; **(b)** dataset sizes stratified by labeling construction process; **(c)** evaluation strategies across studies.

Predictions outcome varied across studies (Figure 5a). While some investigations employed task-specific labels, such as contraindications [52], high-alert drug categories [48], drug-compatibility levels [50], or self-administration events [63], most used binary indicators denoting the presence or absence of a medication error [49, 53, 61, 44, 62, 64, 47, 51, 45, 46, 56]. A final group of studies did not use labels, either because they focused on evaluating existing systems [65, 57, 58, 59, 60] or adopted unsupervised methods [55, 57]. Except for [65], these investigations typically operated on large datasets. In addition, as shown in Figure 5b, relying on expert-annotated labels typically led to smaller datasets than the use of proxy-based or automated labeling strategies.

Most studies formulated the task as a supervised learning problem, predominantly binary classification, in which models separate correct from erroneous medication events [49, 53, 61, 44, 64, 47, 56]. A smaller subset addressed multi-class settings [50, 48]. Several studies combined data-driven learning with predefined clinical rules derived from guidelines or pharmacological knowledge [52, 45]. A number of investigations adopted multi-stage learning pipelines, particularly for unstructured data [62, 63]. These systems decomposed detection into sequential modules, for example, an object, recognition stage to localize medication-related entities or gestures, followed by a temporal classification stage to determine whether an error occurred. Several studies employed unsupervised anomaly-detection approaches [55, 57, 58, 59, 54, 60].

### 3.4 Evaluation strategies and reported outcomes

Evaluation practices varied across studies (Figure 5c), with most relying on a test set [53, 61, 44, 62, 64, 46, 56], and fewer using multiple test sets or cross-validation to assess robustness [47, 52, 63, 55]. Performance evaluation for multi-stage systems was often reported at the module level [62, 46], while anomaly-detection and LLM-based approaches primarily relied on expert review to assess system performance [57, 58, 60, 65]. Prospective or external validation was rare [49, 59, 45].

Reported outcomes mirrored the diversity of evaluation strategies. Supervised classification tasks predominantly rely on standard binary performance metrics such as AUROC or F1-score [49, 53, 61, 62, 64, 47, 56], with AUROC typically ranging from 0.71 to 0.92. Deep-learning systems reported high specificities (≥ 90%) [62, 63] and recalls exceeding 95% in controlled environments [46]. Among unsupervised approaches, precision was consistently high, often exceeding 75% [55, 58, 59, 60]. Recall was not systematically evaluated.

## 4 Discussion

The results reveal two distinct pipelines: one focusing on prescription errors and the other on administration errors. These differ in error type, data modality, modeling strategy, and evaluation context. The prescription pipeline, representing more than two-thirds of included studies, predominantly relies on structured EHR-derived data and tree-based models.

In contrast, administration-focused systems depend on unstructured multimodal inputs (e.g., video, image, or motion signals) and typically employ deep neural networks. Evaluations of administration systems have largely occurred in controlled or simulated environments.

Beyond these two pipelines, we also observe an evolution of training paradigms over time. Early studies relied primarily on anomaly-detection approaches, likely reflecting limited availability of high-quality labels. More recent work increasingly applies tree-based and deep-learning models, paralleling broader developments in clinical ML and expanded access to structured and multimodal data. Notably, LLMs appeared in only one proof-of-concept study [65], indicating that their integration into medication-safety workflows is still widely underexplored.

A recurrent challenge across the included studies was the construction of reliable labels, which often required balancing label quality against dataset size. Several studies derived labels from routine clinical processes, like routine pharmacist reviews [53], dose-related inquiries [44], prescription dose adjustments [47], or voided orders [56]. However, this process introduces inherent labeling inaccuracies. Routine clinical reviews may fail to detect certain errors, while proxy indicators can also be unreliable. For instance, [56] trained their model to predict voided orders, while explicitly noting that approximately 70 ± 10% of these orders correspond to true medication errors. To address these limitations, some investigations relied on expert assessment, with clinicians or pharmacists manually annotating errors through direct observation or detailed chart review. Although this strategy provides higher ground-truth reliability, it is resource-intensive and therefore typically limited to smaller datasets, as underlined in Section 3.3.

Evaluation practices also reflect structural constraints of medication-safety research. Most investigations relied on internal test sets from single-center datasets, limiting insight into cross-institutional performance under varying prescribing cultures and documentation standards. Prospective or external validation was rare, and only a few studies assessed integration within routine workflows. Although reported outcomes were often promising, including one prospective deployment that demonstrated tangible operational benefits, such as reductions in both cost and length of stay [49], the overall evidence base remains fragmented, and the generalizability of current ML approaches remains uncertain.

### 4.1 Implications for practice

ML-based systems for detecting prescription-related medication errors appear to be the most clinically actionable. Owing to the availability of standardized EHR-derived data and their natural alignment with existing medication-review processes, these approaches have received considerable research attention. Consequently, they have been evaluated across a wide range of settings, including several examples of early clinical integration, with consistently promising results.

In contrast, ML methods designed to detect administration errors remain at an earlier stage of development. These systems typically rely on complex multimodal pipelines capable of processing unstructured inputs with substantial associted challenges for dataset construction, scalability, and clinical deployment. These constraints likely explain why most evaluations have occurred in controlled or simulated environments rather than routine clinical practice. While initial findings are encouraging, their real-world feasibility and performance remain uncertain, and these systems should presently be considered experimental prototypes rather than deployable clinical tools.

Finally, most studies were conducted in single-center settings, often with restricted patient populations, limiting the generalizability of their findings. External validation, particularly across institutions with differing prescribing cultures, formularies, and documentation practices, remains scarce. For these reasons, sustained human oversight is essential. ML systems should be viewed not as stand-alone detectors of medication errors but as decision-support tools that augment clinical review, flag high-risk events, and help prioritize pharmacist workload. Institutions aiming for implementation should incorporate rigorous prospective evaluation and establish mechanisms for continuous human verification, feedback, and model updating.

### 4.2 Implications for research

This review identifies several priority areas for advancing research on ML-based medication-error detection.

A first gap concerns medication-administration errors, which remain underexplored and largely evaluated in controlled environments rather than routine workflows. Future work should prioritize close-to-deployment studies assessing workflow integration, human–AI interaction, and alert burden to determine real-world viability.

Research also remains heavily skewed toward structured tabular data. Although a small number of studies leveraged images or other unstructured sources, multimodal approaches remain limited. Notably, LLMs were evaluated in only one proof-of-concept study, underscoring the opportunity to explore their potential for extracting and reasoning over free-text clinical documentation.

A further implication concerns the difficulty of constructing reliable labels. Many studies relied on imperfect proxy indicators, introducing noise and inconsistency. To support real-world deployment, future research should consider methods robust to incomplete or noisy annotations, including noise-robust loss functions [66] and semi-supervised approaches [67].

Despite the well-established impact of hyperparameter optimization on model performance, several studies reported only limited, or in some cases, no, information on how hyperparameters were selected or tuned [33, 45, 63, 44, 55, 46]. In many instances, it was unclear whether any optimization procedure had been applied, and in one study[47] the authors explicitly stated that all models were trained using default settings without conducting hyperparameter tuning. Such incomplete reporting reduces methodological transparency, hinders reproducibility, and may result in inaccurate or biased assessments of model performance. Future research would therefore benefit from systematically documenting the hyperparameter-tuning procedures employed, including the search strategy, parameter ranges, and validation scheme, to support robust comparison across studies and ensure reliable interpretation of results.

Progress is also constrained by the absence of open, standardized benchmarks. Only one publicly available dataset was identified [54], originating from a single center and containing limited prescription attributes. As a result, most evaluations were conducted on isolated datasets under heterogeneous conditions. Developing multicenter, de-identified datasets supported by robust data-sharing frameworks would enable rigorous benchmarking and accelerate methodological advances.

Finally, none of the included studies addressed medication errors in clinical trials. Given the scale of global pharmaceutical research and development and the potential impact of medication errors on data integrity, participant safety, and regulatory compliance [68, 69], this absence is notable. Extending ML-based detection to clinical research settings represents an important opportunity to enhance trial quality and safety.

### 4.3 Strength and limitations of this scoping review

This scoping review has several strengths. It was conducted in accordance with a preregistered protocol and adhered to PRISMA-ScR guidelines, ensuring methodological transparency and reproducibility. A comprehensive search strategy was applied across three major scientific databases, capturing a broad range of ML applications for medication-error detection. Data charting was performed systematically using an extraction framework informed by the CHARMS checklist, enabling consistent assessment of model characteristics, data sources, error types, and evaluation strategies. The review also synthesizes findings across heterogeneous modalities, including structured EHR data, video, sensor signals, images, and free-text inputs, providing a uniquely wide overview of how ML has been applied across the medication-use process.

Despite these strengths, several limitations should be acknowledged. First, the search was restricted to English-language and peer-reviewed publications, which may have led to the omission of relevant studies, particularly emerging work disseminated through preprints or non-English sources. Second, the reporting quality of the included studies varied considerably; key details such as feature definitions, label construction procedures, and data modalities were often incomplete or inconsistently described, limiting direct comparison across studies. Finally, substantial heterogeneity in clinical contexts, data types, and evaluation strategies further constrained the possibility of quantitative synthesis. Consequently, the findings were primarily integrated through narrative synthesis. Despite these limitations, this review provides a comprehensive and structured overview of the current evidence base and highlights clear opportunities for advancing ML research in medication-error detection.

## 5 Conclusion

This scoping review identifies two dominant methodological pipelines for ML-based medication-error detection: a prescription-focused approach grounded in structured EHR data and tree-based models, and a smaller administration-focused paradigm relying on multimodal sensing and deep learning. While reported performance is often promising, the evidence base remains fragmented and largely confined to single-center retrospective evaluations. Label construction strategies vary substantially, external validation is rare, and no standardized benchmarks currently enable fair comparison across models. Beyond routine clinical workflows, no studies addressed medication errors in clinical research settings, despite their distinct operational complexity and safety implications.

Advancing the field will require multicenter datasets, transparent reporting of modeling and tuning procedures, and rigorous prospective evaluation embedded within real-world workflows. Emerging modalities, including free-text processing with large language models, remain underexplored and may offer opportunities to move beyond structured tabular paradigms.

ML systems should therefore be viewed not as autonomous error detectors, but as decision-support tools whose safe and effective deployment depends on robust validation, human oversight, and careful integration into clinical practice.

## Figures and Tables

**Table 1:** List of search terms included in each group.

Group	Search Terms
Group 1	drug error, medication error, contraindication, dosing error, dose error, prescription error, drug administration error, medication administration error, ordering error, route error, frequency error, strength error, formulation error, substitution error, preparation error, transcription error, dispensing error, monitoring error, omission error, commission error, labeling error, documentation error, storage error, wrong patient, wrong drug, wrong dose, wrong route, wrong time, medication safety, medication incident, drug safety
Group 2	drug, medication
Group 3	risk assessment, risk stratification, prediction, detection, classification, categorization
Group 4	artificial intelligence, machine learning, deep learning, neural network, LSTM, long short-term memory, CNN, RNN, GRU, gated recurrent unit, autoencoder, multilayer perceptron, MLP, language model, decision tree, random forest, XGBoost, SVM, support vector machine, gradient boosting, LightGBM, adaptive boosting, AdaBoost, categorical boosting, CatBoost, supervised learning, unsupervised learning, reinforcement learning, ensemble learning, natural language processing, NLP

**Table 2:** Review eligibility criteria based on concept, context, article type, and publication period.

	Inclusion	Exclusion
**Concept**	Studies that develop, apply or evaluate ML methods to predict or detect medication errors.	Studies relying solely on traditional statistical methods or rule-based systems; studies that propose preventive approaches that do not involve medication error predictions.
**Context**	Studies related to clinical treatment or research and involving human subjects.	Studies not related with clinical treatment or research; studies that do not involve human subjects.
**Types of articles**	Basic research articles published in peer-reviewed journals or conference proceedings; full-text available; written in English.	Reviews, editorials, opinion papers, abstracts, posters, dissertations, articles without full text, or not written in English.
**Time period**	Articles published between January 1, 2015 and April 28, 2025.	Articles published before January 1, 2015 or after April 28, 2025.

**Table 3:** Data charting of included studies — **Part A**

Title	Year	Location	Study design	Medication error	Objective	Clinical context	Data source and type
Development and validation of a machine learning model to improve precision prediction for irrational prescriptions in orthopedic perioperative patients [49]	2025	Taiyuan, China	Retrospective with prospective validation study	Prescription errors	Detect irrational prescriptions	Single-center; restricted population	Clinical system; structured data
Using machine learning to predict pharmaceutical interventions during medication prescription review in a hospital setting [53]	2025	Strasbourg, France	Retrospective study	Prescription errors	Evaluate ML models for detecting pharmacist-intervention cases	Single center; non-restricted population	Clinical system; structured data
Unlocking the potential of advanced large language models in medication review and reconciliation: A proof-of-concept investigation [65]	2024	Manama, Bahrain	Simulation-based study	Prescription errors	Evaluate LLMs for identifying medication issues in reconciliation	Artificial environment; non-restricted population	External or derived sources; unstructured data
Machine learning-based risk prediction model for medication administration errors in neonatal intensive care units: A prospective direct observational study [61]	2024	Five cities (name not specified), Malaysia	Prospective study	Administration errors	Predict medication administration errors	Multicenter; restricted population	Clinical system; structured data
Development of machine-learning models using pharmacy inquiry database for predicting dose-related inquiries in a tertiary teaching hospital [44]	2024	Seongnam, South Korea	Retrospective study	Prescription errors	Predict dose-related pharmacy inquiries	Single-center; non-restricted population	Clinical system; structured data
Detecting clinical medication errors with AI enabled wearable cameras [62]	2024	Seattle, USA	Prospective study	Administration errors	Detect vial-swap errors prior	Multicenter; restricted population	Sensors and devices; unstructured data
Development and validation of a machine learning-based detection system to improve precision screening for medication errors in the neonatal intensive care unit [64]	2023	Ankara, Turkey	Prospective study	Prescription, preparation, administration, monitoring errors	Predict medication errors	Single-center; restricted population	Clinical system; structured data
Prediction of Prednisolone Dose Correction Using Machine Learning [47]	2023	Obihiro, Japan	Retrospective study	Prescription errors	Predict dose-related prescription modifications of prednisolone	Single-center; restricted population	Clinical system; structured data
DeepIDC: A Prediction Framework of Injectable Drug Combination Based on Heterogeneous Information and Deep Learning [50]	2022	Chengdu and Beijing, China	Simulation-based study	Contraindication errors	Predict injectable drug incompatibility	Artificial environment; non-restricted population	External or derived sources; structured data
An AI-Empowered Home-Infrastructure to Minimize Medication Errors [51]	2022	Naples, Italy	Simulation-based study	Administration errors	Detect wrong drug	Artificial environment; non-restricted population	Sensors and devices; unstructured data
Use of medication data alone to identify diagnoses and related contraindications: Application of algorithms to close a common documentation gap [52]	2022	Erlangen, Germany	Retrospective study	Prescription error	Detect diagnosis–medication contraindications	Single-center; restricted population	Clinical system; structured data
Hybrid Method Incorporating a Rule-Based Approach and Deep Learning for Prescription Error Prediction [45]	2021	Daejeon, South Korea	Retrospective study	Prescription errors	Detect prescription errors	Single-center; restricted population	Clinical system; structured data
Assessment of medication self-administration using artificial intelligence [63]	2021	Cambridge, USA	Simulation-based study	Administration errors	Detect medication self-administration errors	Artificial environment; restricted population	Sensors and devices; unstructured data
High alert drugs screening using gradient boosting classifier [48]	2021	Chiang Mai, Thailand	Retrospective study	Prescription errors	Detect inappropriate drug prescriptions	Single center; restricted population	Clinical system; structured data
Detection of overdose and underdose prescriptions—An unsupervised machine learning approach [55]	2021	Fukuoka, Japan	Retrospective study	Prescription errors	Detect over/under-dose prescriptions	Single-center; non-restricted population	Clinical system; structured data
Development of a Wearable Camera and AI Algorithm for Medication Behavior Recognition [46]	2021	Seoul, South Korea	Simulation-based study	Medication non-adherence	Detect medication-taking behavior for adherence monitoring	Artificial environment; non-restricted population	Sensors and devices; unstructured data
Predicting self-intercepted medication ordering error using machine learning [56]	2021	Chicago and St. Louis, USA	Retrospective study	Prescription errors	Predict self-intercepted ordering errors	Single-center population; non-restricted population	Clinical system; structured data
Assessing the International Transferability of a Machine Learning Model for Detecting Medication Error in the General Internal Medicine Clinic: Multicenter Preliminary Validation Study [57]	2021	Boston, USA	Retrospective study	Prescription errors	Validate transferability of a ML model	Multicenter; restricted population	Clinical system; structured data
Using a Machine Learning System to Identify and Prevent Medication Prescribing Errors: A Clinical and Cost Analysis Evaluation [58]	2020	Boston, USA	Retrospective study	Prescription errors	Evaluate clinical and economic impact of the MedAware system	Multicenter; non-restricted population	Clinical system; structured data
Reducing drug prescription errors and adverse drug events by application of a probabilistic, machine-learning based clinical decision support system in an inpatient setting [59]	2019	Ramat Gan, Israel	Prospective study	Prescription errors Evaluate clinical impact of the MedAware system	Single center; restricted population	Clinical system; structured data	
DDC-Outlier: Preventing Medication Errors Using Unsupervised Learning [54]	2018	Porto Alegre, Brazil	Retrospective study	Prescription errors	Detect medication-dose and frequency errors	Single-center; non-restricted population	Clinical system; structured data
Screening for medication errors using an outlier detection system [60]	2017	Boston, USA Retrospective validation study		Prescription errors	Evaluate clinical impact of the MedAware system	Multicenter; non-restricted population	Clinical system; structured data

**Table 4:** Data charting of included studies — **Part B**

Ref.	Features	Labels	Dataset size	ML model(s)	Method	Evaluation	Performance
[49]	17 features; patient, prescription, intervention, surgical, clinical and cost characteristics; automatically extracted	Binary labels; expert validated/annotated	3,047 samples; 80/20 train–test split	Tree-based models	Binary classification	Internal test-set evaluation + prospective evaluation	AUROC = 0.92; F1 = 0.71; Recall = 0.80
[53]	14 features; patient, prescription, clinical, and biological characteristics; automatically extracted	Binary labels; routine-workflow-derived	2,059,847 samples; 70/30 train–test split	Tree-based models	Binary classification	Internal test-set evaluation	AUROC = 0.83; F1 = 0.58; Recall = 0.46
[65]	Free-text synthetic clinical scenarios; manually constructed	No labels	18 samples; test set only	LLMs	LLM question-answering / free-text reasoning	Manual expert review	Performance varied by domain with no model consistently superior
[61]	16 features; work environment, medication administration, patient, and provider characteristics; manually collected	Binary labels; expert validated/annotated	1,093 samples; 80/20 train–test split	Tree-based models	Binary classification	Internal test-set evaluation	AUROC = 0.83; F1 = 0.83; Recall = 0.82
[44]	10 features; patient and prescription characteristics; automatically extracted	Binary labels; routine-workflow-derived	20,393 samples; 80/20 train-test split	Tree-based models	Binary classification	Internal test-set evaluation	AUROC = 0.71; AUPRC = 0.89; Recall = 0.92
[62]	Video frames; recorded by anesthesia providers	Binary labels; expert validated/annotated	621 samples; test set of 418 samples	Neural networks	Object detection + rule-based verification	Internal test-set evaluation	Recall = 0.99; Specificity = 0.98
[64]	10 features; patients, pharmacotherapy and providers characteristics; manually collected	Binary labels; routine-workflow-derived	11,908 samples; 70/30 train–test split	Tree-based models	Binary classification	Internal test-set evaluation	AUROC = 0.92; F1 = 0.93; Recall = 0.92
[47]	5 features; patient and prescription characteristics; automatically extracted	Binary labels; routine-workflow-derived	82,553 samples; 70/30 train–test split	Tree-based models	Binary classification	Internal test-set evaluation	AUROC = 0.92; Recall = 0.95; Precision = 0.013
[50]	284 features; chemical and pathway similarity embeddings; automatically generated.	Multiclass labels; expert validated/annotated	3396 samples; 70/30 train-test split; additional dataset of 2496 samples	Neural networks	Multiclass classification	Internal test-set evaluation + unseen-scenario evaluations	AUROC = 0.58 – 0.84; AUPRC = 0.60 – 0.74
[51]	Drug packaging images; manually captured	Binary labels; expert validated/annotated	8,400 samples; 80/20 train-test split	Neural networks	Reinforcement learning-based selection of verification method	Internal test-set evaluation; Qualitative/learning-curve evaluation only for RL agent	Accuracy = 0.98
[52]	Number of features not explicitly discussed; medication presence; automatically extracted	Multiclass labels; expert validated/annotated	3506 samples; 33/66 train-test split	Tree-based models	Rule-based approach integrating classification task	Internal test-set evaluation	Recall = 0.52 – 0.53
[45]	Number not explicitly discussed; patient, prescriptions and clinical characteristics; automatically extracted	Binary labels; expert validated/annotated	139,411 samples; test set of 15,407 samples	Neural networks	Binary classification combined with a rule-based system	Internal test-set performance + external validation	Recall = 0.74 – 0.81; Precision = 0.73–0.76
[63]	Radio-frequency signal reflections of human motion during self-administration; automatically recorded	Multiclass labels; expert validated/annotated	47,788 samples; 80/20 train-test split	Neural networks	Object detection + temporal sequence modeling	5-fold cross-validation	AUROC = 0.91–0.98; Recall = 0.84–0.97; Specificity = 0.92–0.95
[48]	4 features; patient and prescriptions characteristics; automatically constructed	Multiclass labels; routine-workflow-derived	Two datasets of 991,270 and 1,200,000 samples; 75/25 train-test split	Tree-based models	Multi-stage classification + consistency check	Internal test-set performance	Identify 0.98 of mismatch
[55]	3 features; patient and prescriptions characteristics; automatically extracted. 3 features; automatically extracted	No labels; unsupervised learning	21 datasets (between 2022 and 54,423 samples); 80/20 train-test split	Anomaly-detection models	Detection of atypical dosages	5-fold cross-validation	F1 = 0.92 – 0.97; Recall = 0.87 – 0.97; Precision = 0.93 – 0.98
[46]	Image and video data; image resolution not explicitly reported	Binary labels; construction not explicitly discussed	270 samples; 110 samples for test	Neural networks	Object detection + binary classification	Internal test-set performance	Recall = 0.95; Precision = 0.91; Accuracy = 0.93
[56]	13 features; patient, clinician, prescription, date and time characteristics; automatically extracted	Binary labels; routine-workflow-derived	5,804,192 samples; 70/10/20 training, validation and test split	Tree-based models	Binary classification	Internal test-set performance	AUROC = 0.80; AUPRC = 0.06
[57]	Number not explicitly discussed; patient, diagnoses, and prescriptions characteristics; automatically extracted	No labels; unsupervised learning	Two datasets: (i) 1.34 billion samples; (ii) 667,572 samples; test set of 596 samples	Anomaly-detection models	Detection of unsubstantiated prescriptions	Manual expert review	Recall = 0.56 – 0.79; Specificity = 0.87 – 0.93; Accuracy = 0.79 – 0.85
[58]	Number of features not explicitly discussed; patient demographics, encounters, diagnoses, medications, lab results, vital signs, and procedure; automatically extracted	No labels; potential unsupervised approach	Dataset size not reported; tested on 300 samples	Anomaly-detection models	Detection of atypical clinical and dosing patterns	Manual expert review	92% of the alerts were accurate; 79.7% clinical valid
[59]	Number not explicitly discussed; patient, diagnoses, medications, and clinical characteristics; automatically extracted	No labels; potential unsupervised learning	78,017 samples; test-only	Anomaly-detection models	Detection of atypical clinical and dosing patterns	Prospective evaluation	89% of the alerts were accurate; 85% clinically valid; 80% clinically useful
[54]	2 features; medication and prescription characteristics; automatically extracted.	No labels; unsupervised learning	563,171 samples; no train-test split	Anomaly-detection models	Detection of atypical prescriptions	Internal test-set evaluation	F1 = 0.68; Recall = 0.90; Precision = 0.61
[60]	Number not explicitly discussed; patient, diagnoses, clinical, medication, encounter, context characteristics; automaticall extracted	No labels; potential unsupervised learning	Size not reported; tested on 300 samples	Anomaly-detection models	Detection of atypical clinical and dosing patterns	Manual expert review	76.2% of the alerts were clinically valid; among these, 56.2% were high value, 18.8% medium, and 25.0% low

## A Appendix

### A.1 Queries

This appendix provides the exact search queries used for PubMed, Embase, and Web of Science, respectively.

( 
“drug  error*”[Title/Abstract]  OR  “medication  error*”[Title/Abstract]  OR  “contraindication*”[Title/Abstract]  OR  “dosing  error*”[Title/Abstract]  OR  “dose  error*”[Title/Abstract]  OR  “prescri*  error*”[Title/Abstract]  OR  “drug  administrat*  error*”[Title/Abstract]  OR  “medication  administrat*  error*”[Title/Abstract]  OR  “order*  error*”[Title/Abstract]  OR  “route  error*”[Title/Abstract]  OR  “frequency error*”[Title/Abstract]    OR    “strength    error*”[Title/Abstract]    OR    “formulation    error*”[Title/Abstract ]  OR  “substitution  error*”[Title/Abstract]  OR  “preparation  error*”[Title/Abstract]  OR  “transcrib* error*”[Title/Abstract]    OR    “dispensing    error*”[Title/Abstract]    OR    “monitoring    error*”[Title/Abstract]  OR  “omission  error*”[Title/Abstract]  OR  “commission  error*”[Title/Abstract]  OR  “labeling error*”[Title/Abstract]  OR  “documentation  error*”[Title/Abstract]  OR  “storage  error*”[Title/Abstract]  OR  “wrong  patient”[Title/Abstract]  OR  “wrong  drug”[Title/Abstract]  OR  “wrong  dose”[Title/Abstract]  OR  “wrong  route”[Title/Abstract]  OR  “wrong  time”[Title/Abstract]  OR  “medication  safety”[Title/Abstract]  OR  “medication  incident*”[Title/Abstract]  OR  “drug  safety”[Title/Abstract] 
) 
AND 
(
“drug”[Title/Abstract]    OR    “drugs”[Title/Abstract]    OR    “medication*”[Title/Abstract] 
) 
AND 
( 
“risk  assessment”[Title/Abstract]  OR  “risk  stratification”[Title/Abstract]  OR 
“prediction”[Title/Abstract]  OR  “detection”[Title/Abstract]  OR  “classif*”[Title/Abstract]  OR  “ 
categor*”[Title/Abstract] 
) 
AND 
( 
“artificial  intelligence”[Title/Abstract]  OR  “machine  learning”[Title/Abstract]  OR  “deep  learning”[Title/Abstract]  OR  “neural  network*”[Title/Abstract]  OR  “LSTM”[Title/Abstract]  OR  “long  short-term memory”[Title/Abstract] OR  “CNN”[Title/Abstract] OR  “RNN”[Title/Abstract] OR  “GRU”[Title/Abstract] OR  “gated  recurrent  unit*”[Title/Abstract]  OR  “autoencoder*”[Title/Abstract]  OR  “multilayer perceptron*”[Title/Abstract]  OR  “MLP”[Title/Abstract]  OR  “language  model*”[Title/Abstract]  OR  “ decision  tree*”[Title/Abstract]  OR  “random  forest*”[Title/Abstract]  OR  “XGBoost”[Title/Abstract]  OR “SVM”[Title/Abstract]  OR  “support  vector  machine”[Title/Abstract]  OR  “gradient  boosting”[Title/Abstract]  OR  “LightGBM”[Title/Abstract]  OR  “adaptive  boosting”[Title/Abstract]  OR  “AdaBoost”[Title/Abstract]  OR  “categorical  boosting”[Title/Abstract]  OR  “CatBoost”[Title/Abstract]  OR  “supervised learning”[Title/Abstract]  OR  “unsupervised  learning”[Title/Abstract]  OR  “reinforcement  learning”[Title/Abstract]  OR  “ensemble  learning”[Title/Abstract]  OR  “natural  language  processing”[Title/Abstract]  OR  “NLP”[Title/Abstract] 
) 
AND  (english[Filter])  AND  (2015:2025[pdat]) 
(
  ‘drug error*’:ab,ti OR ‘medication error*’:ab,ti OR ‘contraindication*’:ab,ti OR 
  ‘dosing error*’:ab,ti OR ‘dose error*’:ab,ti OR ‘prescri* error*’:ab,ti OR
  ‘drug administrat* error*’:ab,ti OR ‘medication administrat* error*’:ab,ti OR ‘order* error*’:ab,
  ti OR
  ‘route error*’:ab,ti OR ‘frequency error*’:ab,ti OR ‘strength error*’:ab,ti OR 
  ‘formulation error*’:ab,ti OR ‘substitution error*’:ab,ti OR ‘preparation error*’:ab,ti OR 
  ‘transcrib* error*’:ab,ti OR ‘dispensing error*’:ab,ti OR ‘monitoring error*’:ab,ti OR 
  ‘omission error*’:ab,ti OR ‘commission error*’:ab,ti OR ‘labeling error*’:ab,ti OR 
  ‘documentation error*’:ab,ti OR ‘storage error*’:ab,ti OR ‘wrong patient’:ab,ti OR 
  ‘wrong drug’:ab,ti OR ‘wrong dose’:ab,ti OR ‘wrong route’:ab,ti OR
  ‘wrong time’:ab,ti OR ‘medication safety’:ab,ti OR ‘medication incident*’:ab,ti OR ‘drug safety’: 
  ab,ti
)
AND
(
  ‘drug*’:ab,ti OR ‘medication*’:ab,ti
)
AND
(
 ‘risk assessment’:ab,ti OR ‘risk stratification’:ab,ti OR
 ‘prediction’:ab,ti OR ‘detection’:ab,ti OR ‘classif’:ab,ti OR ‘categor*’:ab,ti
)
AND
(
  ‘artificial intelligence’:ab,ti OR ‘machine learning’:ab,ti OR ‘deep learning’:ab,ti OR 
  ‘neural network*’:ab,ti OR ‘LSTM’:ab,ti OR ‘long short-term memory’:ab,ti OR ‘CNN’:ab,ti OR ‘RNN’: 
  ab,ti OR
  ‘GRU’:ab,ti OR ‘gated recurrent unit*’:ab,ti OR ‘autoencoder*’:ab,ti OR ‘multilayer perceptron*’: 
  ab,ti OR
  ‘MLP’:ab,ti OR ‘language model*’:ab,ti OR ‘decision tree*’:ab,ti OR 
  ‘random forest*’:ab,ti OR ‘XGBoost’:ab,ti OR ‘SVM’:ab,ti OR
  ‘support vector machine’:ab,ti OR ‘gradient boosting’:ab,ti OR ‘LightGBM’:ab,ti OR ‘adaptive 
  boosting’: ab,ti
OR ‘AdaBoost’:ab,ti OR ‘categorical boosting’:ab,ti OR ‘CatBoost”:ab,ti 
OR ‘supervised learning’:ab,ti OR 
  ‘unsupervised learning’:ab,ti OR ‘reinforcement learning’:ab,ti OR 
  ‘ensemble learning’:ab,ti OR ‘natural language processing’:ab,ti OR ‘NLP’:ab,ti
)
AND [english]/lim 
AND [2015–2025]/py
(
  “drug error*” OR “medication error*” OR “contraindication*” OR
  “dosing error*” OR “dose error*” OR “prescri* error*” OR
  “drug administrat* error*” OR “medication administrat* error*” OR “order* error*” OR
  “route error*” OR “frequency error*” OR “strength error*” OR
  “formulation error*” OR “substitution error*” OR “preparation error*” OR
  “transcrib* error*” OR “dispensing error*” OR “monitoring error*” OR
  “omission error*” OR “commission error*” OR “labeling error*” OR
  “documentation error*” OR “storage error*” OR “wrong patient” OR
  “wrong drug” OR “wrong dose” OR “wrong route” OR
  “wrong time” OR “medication safety” OR “medication incident*” OR “drug safety”
)
AND
(“drug*” OR “medication*”)
AND
(
  “risk assessment” OR “risk stratification” OR
 “prediction” OR “detection” OR “classif*” OR “categor*”
)
AND
(
  “artificial intelligence” OR “machine learning” OR “deep learning” OR
  “neural network*” OR “LSTM” OR “long short-term memory” OR “CNN” OR “RNN” OR
  “GRU” OR “gated recurrent unit*” OR “autoencoder*” OR “multilayer perceptron*” OR
  “MLP” OR “language model*” OR “decision tree*” OR
  “random forest*” OR “XGBoost” OR “SVM” OR
  “support vector machine” OR “gradient boosting” OR “LightGBM” OR “adaptive boosting” OR “AdaBoost
  “ OR “categorical boosting” OR “CatBoost”
OR “supervised learning” OR
  “unsupervised learning” OR “reinforcement learning” OR
  “ensemble learning” OR “natural language processing” OR “NLP”
)

### A.2 Extraction table

**Table 5:** Description of the information extracted from each included study.

Item	Information extracted
Title	Full title of the article.
Author(s)	Name of the first author.
Year	Year of publication.
Journal/Conference	Name of the journal or the conference where the article has been published.
Location	Geographic location where the study was conducted (if reported).
Study design	Type of study (e.g., retrospective study, prospective study, validation study, feasibility study).
Medication errors	Type of medication errors considered in the study (e.g., administration errors, prescription errors, dosing errors).
Study objective	Objective of the study (e.g., predicting administration errors, detecting prescribing errors).
Clinical context	Clinical setting and environment (e.g., hospital, outpatient clinic, pharmacy), therapeutic area or disease focus, target population characteristics (e.g., age group, risk profile), and clinical intervention or research.
Data sources and type	Origin of data (e.g., electronic health records, registry, clinical trial, claims), and data modality (e.g., structured data, medical imaging, free-text clinical notes, multi-modal).
Features	Description of the features and their construction process.
Labels	Description of the labels and their annotation process.
Dataset size	Number of data instances for training, validation, and testing, including any external datasets if applicable.
ML model(s)	ML technique(s) used (e.g., decision trees, random forests, LSTM, large language models).
Method	Description of how ML was used to predict or detect medication errors; specific task formulation (e.g., classification, regression, anomaly detection); integration into the clinical workflow if reported.
Evaluation method	Description of validation strategies (e.g., cross-validation, test set, external validation).
Performance	Reported performance metrics (e.g., accuracy, AUROC, precision, recall, F1-score).
Additional notes	Any other relevant information or observations not captured by the categories above.
